# Acoustic Denoising Using Artificial Intelligence for Wood-Boring Pests *Semanotus bifasciatus* Larvae Early Monitoring

**DOI:** 10.3390/s22103861

**Published:** 2022-05-19

**Authors:** Xuanxin Liu, Haiyan Zhang, Qi Jiang, Lili Ren, Zhibo Chen, Youqing Luo, Juhu Li

**Affiliations:** 1 School of Information Science and Technology, Beijing Forestry University, Beijing 100083, China; liuxuanxin@bjfu.edu.cn (X.L.); zhyzml@bjfu.edu.cn (H.Z.); lijuhu@bjfu.edu.cn (J.L.); 2 Engineering Research Center for Forestry-Oriented Intelligent Information Processing of National Forestry and Grassland Administration, Beijing 100083, China; 3 Beijing Key Laboratory for Forest Pest Control, Beijing Forestry University, Beijing 100083, China; jiangqi@bjfu.edu.cn (Q.J.); lily_ren@bjfu.edu.cn (L.R.); yqluo@bjfu.edu.cn (Y.L.)

**Keywords:** acoustic denoising, *Semanotus bifasciatus*, feeding sounds, artificial intelligence, time domain, frequency domain

## Abstract

Acoustic detection technology is a new method for early monitoring of wood-boring pests, and the effective denoising methods are the premise of acoustic detection in forests. This paper used sensors to record *Semanotus bifasciatus* larval feeding sounds and various environmental noises, and two kinds of sounds were mixed to obtain the noisy feeding sounds with controllable noise intensity. Then, the time domain denoising models and frequency domain denoising models were designed, and the denoising effects were compared using the metrics of a signal-to-noise ratio (SNR), a segment signal-noise ratio (SegSNR), and log spectral distance (LSD). In the experiments, the average SNR increment could achieve 17.53 dB and 11.10 dB using the in the test data using the time domain features and frequency domain features, respectively. The average SegSNR increment achieved 18.59 dB and 12.04 dB, respectively, and the average LSD between pure feeding sounds and denoised feeding sounds were 0.85 dB and 0.84 dB, respectively. The experimental results demonstrated that the denoising models based on artificial intelligence were effective methods for *S*. *bifasciatus* larval feeding sounds, and the overall denoising effect was more significant, especially at low SNRs. In view of that, the denoising models using time domain features were more suitable for the forest area and quarantine environment with complex noise types and large noise interference.

## 1. Introduction

Forest biological disasters caused by forest pests were one of the major natural disasters threatening forestry development. Their damage to forest resources and ecosystems caused a large number of direct or indirect economic losses every year [1,2]. In forest pests, wood-boring pests were particularly difficult to control because of their hidden life and lagging victimization.

At present, the commonly monitoring methods of pests mainly include manual sample plot observation, adult trapping technology, aerospace remote sensing monitoring, and so on. Traditional manual observation has problems such as low efficiency, high labor intensity, and destructive dissection. Adult trapping technology has the advantages of strong luring power and high specificity, etc. However, that method only monitors adult pests, while the damage of wood-boring pests is more serious in the larval stage. Remote sensing monitoring could achieve large-scale monitoring but relies on visual features of forest trees, and the early warning of pest situations is more delayed.

In recent years, with the development of computer and sensor technology and the decrease in sensor cost, acoustic detection technology has been gradually widely applied to the field of pest monitoring [1,3,4]. Acoustic detection technology could collect the activity signals of pests in the trunk with the help of piezoelectric sensors and other equipment and promptly warn in the early stage of pests, which has a broad application prospect in the monitoring of wood-boring pests [5,6,7].

For adult pests with vocal organs or larval pests in a sound insulation environment, microphones were usually used as sensors to listen for the vibration transmitted to the air. Luo et al. used an SM4001 microphone (BSWA TECH Inc., Beijing, China) to record the stridulation signals of four bark beetles [8]. Njoroge et al. used the 0.5″ microphone (Model 378B02, PCB Poezotonics Inc., Depew, NY, USA) to record the movement and feeding activities of *Prostephanus truncatus* and *Sitophilus zeamais* [9]. In order to record the signals with a high-frequency rate, Avisoft Bioacoustics CM16/CMPA ultrasound microphones (Avisoft Bioacoustics e.K., Glienicke/Nordbahn, Germany) also have been used for sunn pests [10,11]. Although the microphone sensor had a low price, high sensitivity, and wide frequency response range, it was easy to be disturbed by environmental noises, and it mainly received vibration in the air and could not directly sense vibration in the trunk. Thus, the piezoelectric sensors were used to record the activity signals of pests. The pickup with a piezoelectric transducer as a sensing element was embedded in the trunk and recorded the vibration signals generated by the activities of wood-boring pests. Herrick and Mankin used an AED-2000 amplifier (AEC Inc., Monrovia, CA, USA) with a Model SP-1L sensor-preamplifier module to record the sounds of *Rhynchophorus Ferrugineus* [12]. Bu et al. used AED 2010L (AEC Inc., Monrovia, CA, USA), including a piezoelectric sensor SP-1L and a portable acoustic emission amplifier, to record four types of acoustic behaviors of *Anoplophora glabripennis* and *Anoplophora chinensis* [5].

At present, the research of pest acoustic signals mainly focuses on the analysis of the behavior of pests [5,13,14,15], population density estimation [16], and acoustic signal recognition of pests [17,18,19]. Most of the research data were pure sounds collected in a sound insulation chamber [20] or indoor environment. However, the acoustic detection technology of wood-boring pests would face complex environmental noises in practical application, which would greatly interfere with the subsequent analysis and identification. Some researchers have considered the problem of noise interference. For some data with noises in the acquisition process, the noises were usually filtered manually [21,22]. Some researchers have also considered the superposition of vibrations coming from feeding sounds and environmental noises and extracted the features of noisy feeding sounds to improve the robustness of the classifier [19,23,24]. However, when the sound’s data were large, the manual filter would face the problems of a heavy workload, low efficiency, and so on, and the above solutions were only applicable to the environment with low noise intensity, and the recognition accuracy decreased to less than 90% when the signal-to-noise ratio (SNR) of the noisy feeding sounds at −7 dB [19]. Therefore, effective denoising methods were of great significance and value to the research and analysis of acoustic signals and the practical application of acoustic detection technology.

In the existing research, the denoising methods of acoustic signals could be divided into two categories: traditional denoising methods and artificial intelligence denoising methods. Traditional methods, such as spectral subtraction [25], wavelet transform [26,27], discrete cosine transform [28], Non-negative Matrix Factorization (NMF) [29], Empirical Mode Decomposition (EMD) [30], and so on, were based on the assumed statistical properties of noise signals and required subjective threshold setting. The denoising method based on local mean decomposition (LMD) and wavelet packet denoising were used to denoise the vibration signal of a diaphragm pump. However, the threshold of frequency components filtering needs to be determined manually [31], and the EMD threshold denoising method optimized by an improved fruit fly optimization algorithm (IFOA) was launched to eliminate noise components from machinery sound but the simulation experiment used only Gaussion white noise without quantitative testing of more complex environmental noise [32]. The Go decomposition (Godec) based on the robust principal component analysis (RPCA) had been used to denoised the underwater acoustic signal [33]. This method was mainly applicable to low-rank signals or approximately low-rank signals, which may drop drastically when the signals are complex. Image denoising [34] and acoustic denoising were similar to some extent, so some methods in the field of image denoising can also be migrated to acoustic signals. The guided filter based on wavelet transform was proposed to denoise the sonar signal. However, the simulation experiment still used only Gaussion noises considering the distribution of ocean noise [35]. Most of traditional denoising methods only dealt with a signal type of noise. However, the noise that needed to be dealt with in real situations may be more complex, more irregular, or just unwanted sounds in the current issue. On the basis of that, artificial intelligence denoising methods, which could automatically and flexibly handle different types of noise, increasingly used in the field of acoustic denoising especially in the field of speech denoising. Speech denoising, also called speech enhancement, could be further divided into frequency domain denoising methods and time domain denoising methods. Frequency domain denoising methods calculated the frequency spectrograms as the input of neural network. Then the neural network extracted the frequency domain features to generate the spectrograms for denoised audio. Finally, the spectrograms were converted to the waveform of audio and the audio files were exported [36,37,38]. U-Net architecture [39,40] and convolutional neural network-based generative adversarial network (CNN-GAN) all had been used for speech enhancement in the frequency domain. Except that, LSTM architecture was used in the field of speech separation, which could be seen as a more complex denoising scene [41]. The time domain denoising methods directly used the waveform information to extract the time domain features for denoising. The methods needed to process a large number of data points but did not need spectrum conversion before and after denoising [42]. In order to strengthen the feature association between contexts, dilated convolutions were often used to extract time domain features [43,44]. In addition, multi-domain processing via hybrid denoising networks (MDPhD) was proposed, which improved the performance of speech enhancement by integrating the information of time domain and frequency [45]. With the development of speech denoising technology based on artificial intelligence, it has been used in electrocardiogram (ECG) signal denoising [46,47], cab signal denoising [48], sonar [49], and other fields in recent years.

*Semanotus bifasciatus* Motschulsky (Coleoptera: Cerambycidae) is one of the wood-boring pests that endanger *Platycladus* trees. This study recorded the *S. bifasciatus* larval feeding sounds and various environmental noises to construct the noisy feeding sounds with controllable noise intensity. Then the feeding sounds denoising models based on artificial intelligence were designed from the perspective of the frequency domain and time domain, respectively. Through a comprehensive analysis of the denoising effects for the above models, this study could provide a denoising scheme for acoustic detection so as to lay a foundation for early acoustic detection of wood-boring pests in forests. The main contributions of this paper were summarized as follows:

(1) The piezoelectric sensor connected with a voltage collection module was used to record the feeding sounds of *Semanotus bifasciatus* larvae.
(2) The time domain denoising models based on the standard convolutions and dilated convolutions were designed to denoise the noisy feeding sounds according to the waveforms.
(3) The frequency domain denoising models based on different recurrent layers were designed to denoise the noisy feeding sounds according to the spectrograms.
(4) The denoising effects of different denoising models were evaluated and compared from the perspective of waveform and spectrum.

The rest of this study is organized as follows: Section 2 introduces the dataset construction. Section 3 explains the network architecture of the designed denoising models. Section 4 presents the experiments and results. Section 5 discusses the denoising effects and the advantages and limitations of the denoising models. Finally, Section 6 provides the conclusions of the study.

## 2. Data Materials

### 2.1. Data Recording

In this study, ten non-infested logs of *Platycladus orientalis* woods (length 30 cm, diameter 10 cm) were prepared for insect infestation, and the signal waveguide screw (length 76 mm, diameter 1.6 mm) was inserted in the middle of each log at a depth of 3 cm. Five pairs of *S. bifasciatus* adults were inoculated in each log in mid and late March, waiting for the adults to mate and oviposit on logs.

**Figure 1:**
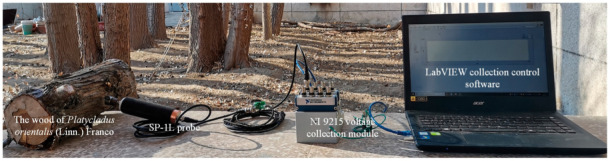
The instruments for data recording.

In April and May, the piezoelectric sensor probe SP-1L (AEC Inc., Monrovia, CA, USA) connected with NI 9215 voltage collection module (NI Inc., Austin, TX, USA) was used to record the feeding sounds of *S. bifasciatus* larvae in the wood every 3 to 5 days and the collection process covered the entire life cycle from first instar larvae to old mature larvae. The instruments for data recording are shown in Figure 1.

When *S. bifasciatus* larvae fed, the mouthparts rubbed against wood and produced vibrations, and then the vibration signals were transmitted inside the solid through the drill to the piezoelectric ceramic sensor inside the probe. The mechanical energy was converted to an electrical signal by the sensor. After passing through the amplifier and the AC/DC separation circuit, the electrical signal was captured with a sample rate of 44.1 kHz and a sampling depth of 32 bits by voltage collection module and stored in the computer in .tdms format. Finally, the .tdms format data were converted to mono .wav format using LibVIEW.

**Figure 2:**
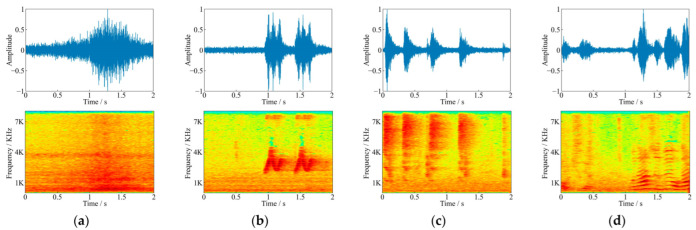
Comparison of waveforms and spectrograms for different kinds of environmental noise, (**a**) vehicle driving, (**b**) whistle, (**c**) bird singing, and (**d**) human voice.

After recording, the infested log was stripped to count the number of larvae. The average oviposition of females was 72.54 ± 22.13, and the hatching rate was 87.25%. The feeding sounds recorded in the above experiment were pure feeding sounds without environmental noises. In order to carry out denoising research, a non-infested log was placed in noisy environment such as grove and roadside to record environmental noises using the same instruments. The recorded noises included obvious noises such as vehicle driving, whistle, bird singing, wind, dripping sound, human voice, and so on (as shown in Figure 2).

In the experiment, the recorded feeding sounds and environmental noises were divided into audio segments with a length of 5 min per segment, and the sampling rate was 44.1 kHz. Then 60 audio segments were selected discontinuously for each type to study, and the sampling rate was converted to 16.0 kHz and 16 bit using Sound eXchange (Sox) audio processing tool.

### 2.2. Dataset Construction

This study used 84 audio segments for training and 36 audio segments for test in all. In order to facilitate the training of denoising models, the audio segments were further divided into audio slices with a length of 2 s. After slicing, there were 12,600 slices in training set and 5400 slices in test set (as shown in Table 1).

In order to obtain the noisy feeding sounds with controllable noise intensity, the pure feeding sound slices and environmental noises were mixed with a certain noise intensity [50], and the noise intensity was measured by the SNR [51], which was defined as follows:

(1) $$Sig_{SNR}=10\times \log_{10}\left[\frac{\sum_{t=1}^{n}C_{t}{}^{2}}{\sum_{t=1}^{n}{\left(S_{t}-C_{t}\right)}^{2}}\right]$$

where $C_{t}$ was the amplitude of pure feeding sound at the *t*-th sampling point, $S_{t}$ was the amplitude of noisy feeding sound at the *t*-th sampling point, and *n* was the number of sampling points for the audio.

**Figure 3:**
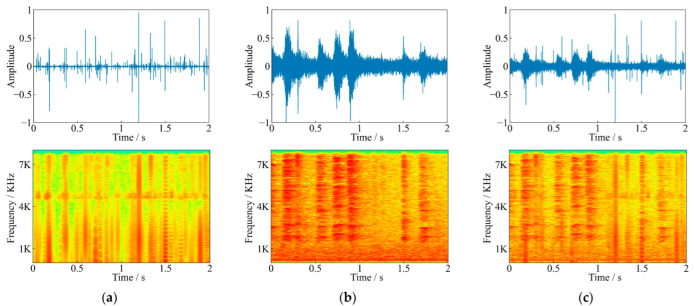
Comparison of waveforms and spectrograms before and after mixing (**a**) *S. bifasciatus* feeding sound, (**b**) noise audio containing bird songs, (**c**) mixed audio at −5 dB SNR.

There were 6 SNRs (−20 dB to 5 dB, with an interval of 5 dB) used for the mixing of the training set in the experiments. Each pair of pure feeding sounds and environmental noises were mixed at one of the above SNRs randomly. Finally, there were 1050 mixing noisy feeding sounds for each SNRs, with a total of 6300 noisy feeding sounds in the training set. Figure 3 shows an example of the waveforms and frequency spectrograms before and after mixing. There were clear biting pulses in the spectrograms of pure feeding sound. However, these pulses in the mixed audio were no longer apparent, and the spectral features of bird songs emerged after superimposing, which was more likely to the data recorded in noisy forest. Thus, the purpose of denoising was to strip the noise from the noisy feeding sounds and regain pure feeding sounds, laying the foundation for further analysis and research.

The mixing mode of the test set was basically same as that of the training set. In order to better test the denoising effects of the methods, the range of mixing SNRs was expanded for the test set. There were still 6 SNRs, but the range was from −24 dB to 6 dB (with an interval of 6 dB). Finally, there were 450 mixing noisy feeding sounds for each SNR, with a total of 2700 noisy feeding sounds in the test set.

## 3. Denoising Models Based on Artificial Intelligence

### 3.1. Time Domain Denoising Networks

In this study, time domain denoising networks (TDD-Nets) were the artificial intelligence denoising models designed to extract the time domain features for the denoising of noisy feeding sounds. The unit of the waveform was a sampling point, and a 1 s piece of audio contained tens of thousands of sample points. Since the waveform of audio contained a high density of data samples and a low-value density of audio features, the dilated convolutions [52], which could effectively increase the receptive field of the model, were more suitable for the denoising in the time domain compared with the standard convolutions. Dilated convolutions added the concept of dilated on the basis of standard convolution. It expanded the coverage of the convolution kernel by setting the dilated rate.

**Figure 4:**
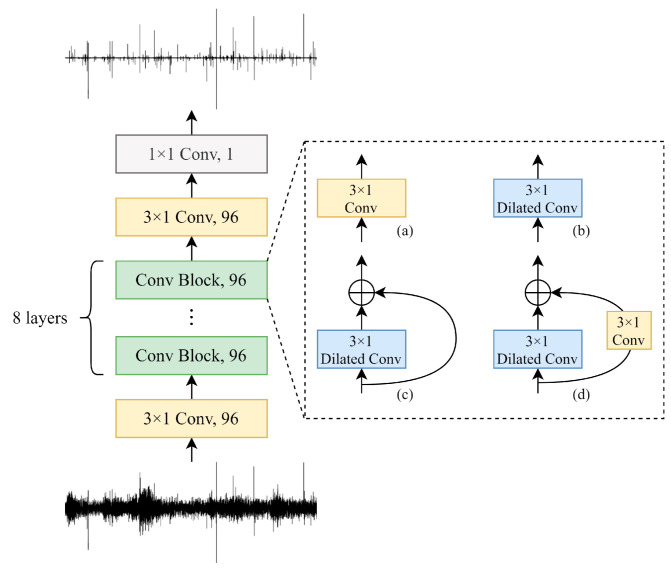
The framework of TDD-Nets with different convolution blocks. (**a**) The standard convolution. (**b**) The dilated convolution. (**c**) The dilated convolution with a shortcut. (**d**) The dilated convolution with a convolutional shortcut.

The framework of the proposed TDD-Nets is shown in Figure 4. The networks consisted of three standard convolutional layers and eight convolution blocks. The convolution blocks used three different dilated convolution structures and are compared with the standard convolutions. The four variants of TDD-Nets were TDD-Nets using convolution (TDD-Nets-C) (Figure 4a), TDD-Nets using dilated convolution (TDD-Nets-D) (Figure 4b), TDD-Nets using dilated convolution with a shortcut (TDD-Nets-DS) Figure 4c) and TDD-Nets using dilated convolution with a convolutional shortcut (TDD-Nets-DC) (Figure 4d) respectively.

In this study, TDD-Nets-C was the standard convolutional network. TDD-Nets-D replace the standard convolutions with dilated convolutions in the convolution blocks in order to learn the multiscale correlation between non-adjacent data more effectively. TDD-Nets-DS added a shortcut on the basis of TDD-Nets-D in order to improve the gradient dissipation problem, and TDD-Nets-DC added convolutions in the shortcut to extract some local correlation between adjacent data.

In order to obtain the front and back waveform information in the time domain more widely, the dilated rate of dilated convolutions in the model was increased from 2^1^ to 2^8^ layer by layer; that is, the dilated rate was 2*^k^* in the *k*-th convolution block, which used the dilated convolutions. The batch normalization (BN) layer and the leaky rectified linear unit (ReLU) activation function were added after every standard convolution and dilated convolutions in the model except the last 1 × 1 convolution, in which the former was used to maintain the consistency of input data distribution at each layer of the network, and the latter was used to realize the nonlinear mapping of the model.

The loss function of the TDD-Nets was the mean square error (MSE). MSE was defined as an average of the square of the difference between actual and estimated values. In this experiment, it was computed as follows:

(2) $$Loss=\frac{\sum_{t=1}^{n}{\left(C_{t}-D_{t}\right)}^{2}}{n}$$

where $D_{t}$ was the amplitude of denoised feeding sound.

### 3.2. Frequency Domain Denoising Networks

In this study, frequency denoising networks (FDD-Nets) were the artificial intelligence denoising models designed to extract the frequency domain features for the denoising of noisy feeding sounds. The time–frequency masking [53] was the common method of denoising using frequency features, which generated a masking matrix and constructed the denoised feeding sound according to the masking matrix:

(3) $$F_{denoised}=F_{noisy}\times M$$

where $F_{denoised}$ was the spectrogram for denoised feeding sound, $F_{noisy}$ was the spectrogram for noisy feeding sound, and *M* was the masking matrix.

As the spectrogram was the splicing of audio frequency characteristics in frames, the context frames had high correlation and time dependence. So the recurrent layers [54], which could correlate context features and effectively solve the problems related to sequence data, were more suitable for the feature extraction of frequency domain features.

**Figure 5:**
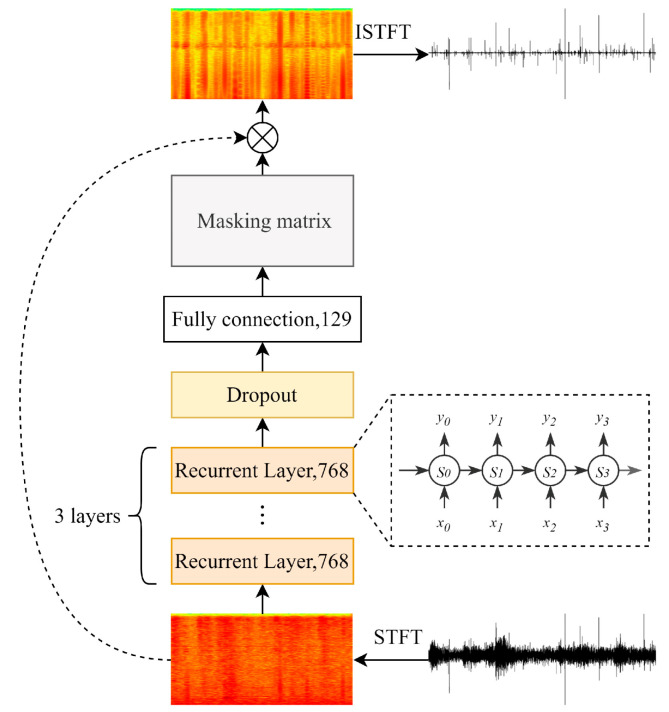
The framework of FDD-Nets.

The framework of the proposed FDD-Nets is shown in Figure 5. The networks consisted of three recurrent layers and a fully connected layer with ReLU activation. The recurrent layers were used to learn frequency domain features frame by frame, and the full connection was used to map the frame dimension in the frequency domain and generate a masking matrix consistent with the size of the spectrogram, and the dropout layer was used before the fully connected layer to increase the randomness of the model and avoid overfitting. Further, this study compared the effects of six kinds of recurrent layers, which were recurrent neural network (RNN) [54], gated recurrent unit (GRU) [55], long short-term memory (LSTM) [56], bidirectional recurrent neural networks (BRNN) [57], bidirectional gated recurrent unit (BiGRU) and bidirectional long short-term memory (BiLSTM), and the corresponding FDD-Nets were called FDD-Nets using RNN (FDD-Nets-R), FDD-Nets using GRU (FDD-Nets-G), FDD-Nets using LSTM (FDD-Nets-L), FDD-Nets using BRNN (FDD-Nets-BR), FDD-Nets using BiGRU (FDD-Nets-BG), FDD-Nets using BiLSTM (FDD-Nets-BL) respectively.

RNN was a sequence-based structure that established connections between current moments and previous moments in a sequence and extracted features from the sequence itself and the connections between moments [54]. However, it was limited to the length of the sequence for which the RNN was able to establish connections. Thus, LSTM was proposed to solve the long-term dependency of RNN. It added three gates called input gate, forget gate, and output gate in the RNN unit to optionally retain information from previous moments in order to protect and control the cell state [56]. GRU was a variation on LSTM, which only used two gates called reset gate and update gate to implement similar functions to LSTM [55]. Standard recurrent layers only focused on information from previous moments, but the information from future moments was equally valuable. Thus, the BRNN was proposed to consolidate all available input information in the past and future of a specific moment [57], and BiGRU and BiLSTM were proposed based on the same thought.

In order to obtain the spectrogram of the feeding sounds, the short-time Fourier transform (STFT) was used with Hann windowing size of 256 samples and hop size of 128 samples. The generated spectrograms were 129 × 249 pixels for each audio with the length of 2 s, and the denoised spectrograms generated by FDD-Nets were converted into waveforms by inverse short-time Fourier transform (ISTFT).

The loss function of the FDD-Nets was also the MSE, which was computed as follows:

(4) $$Loss=\frac{\sum {\left(F_{noisy}\times \hat{M}-F_{pure}\right)}^{2}}{N}$$

where $F_{pure}$ was the spectrogram for pure feeding sound, $\hat{M}$ was the masking matrix the FDD-Nets generated, and *N* was the number of points in the spectrograms.

## 4. Experiments and Results

### 4.1. Implementation Details

In this study, all of the models based on artificial intelligence were implemented in PyTorch deep learning framework and run on the workstation with one AMD Ryzen Threadripper 3990X Processor (128 GB memory) (Advanced Micro Devices, Inc., Santa Clara, CA, USA) and one NVIDIA GeForce RTX 3090 GPU (24 GB graphic memory) (NVIDIA Corporation, Santa Clara, CA, USA).

The models were trained for 50 epochs on each mini-batch with a batch size of 16, and the Adam algorithm was used as the optimizer. The initial learning rate of the TDD-Nets was 0.001, and it was decayed with an attenuation factor of 0.5 every 20 epochs, and the initial learning rate of the FDD-Nets was 0.0001, and it was updated through dynamic attenuation that the learning rate was decayed with an attenuation factor of 0.7 if the training loss did not decrease.

### 4.2. Evaluation Metrics for Denoising

In this study, SNR, segmental signal-noise ratio (SegSNR) [58], and log spectral distance (LSD) [59] were used to evaluate the quality of the denoised feeding sounds of *S. bifasciatus* larvae. The SNR and SegSNR evaluated the denoising effects from the perspective of the whole and local signal strength of noises after denoising, respectively, and the LSD evaluated the denoising effects from the perspective of spectral similarity of denoised feeding sounds and pure feeding sounds.

SNR meant the ratio of pure feeding sound energy to noise audio energy. In order to better show the effectiveness of the denoising models, ∆SNR was used to represent the increment of SNR after denoising, and the larger ∆SNR, the better the denoising effect. The equation of ∆SNR was shown as follows:

(5) $$\Delta Sig_{SNR}={\left(Sig_{SNR}\right)}_{\text{before denoising}}-{\left(Sig_{SNR}\right)}_{\text{after denoising}}$$

(6) $${\left(Sig_{SNR}\right)}_{\text{before denoising}}=10\times \log_{10}\left[\frac{\sum_{t=1}^{n}C_{t}{}^{2}}{\sum_{t=1}^{n}{\left(S_{t}-C_{t}\right)}^{2}}\right]$$

(7) $${\left(Sig_{SNR}\right)}_{\text{after denoising}}=10\times \log_{10}\left[\frac{\sum_{t=1}^{n}C_{t}{}^{2}}{\sum_{t=1}^{n}{\left(D_{t}-C_{t}\right)}^{2}}\right]$$

SegSNR meant the average value of the SNR calculated by frame. As the feeding sounds were mainly short-term continuous, SegSNR could better reflect the local difference of sounds, and ∆SegSNR meant the increment of SegSNR after denoising, which was shown as follows:

(8) $$\Delta Sig_{SegSNR}={\left(Sig_{SegSNR}\right)}_{\text{before denoising}}-{\left(Sig_{SegSNR}\right)}_{\text{after denoising}}$$

(9) $${\left(Sig_{SegSNR}\right)}_{\text{before denoising}}=\frac{\sum_{i=1}^{f}{\left(Sig_{SNR}\right)}_{\text{before denoising}}^{f}}{f}$$

(10) $${\left(Sig_{SegSNR}\right)}_{\text{after denoising}}=\frac{\sum_{i=1}^{f}{\left(Sig_{SNR}\right)}_{\text{after denoising}}^{f}}{f}$$

where f was the frame number in the audio. In this paper, the windowing size was 256 samples, and the shift size was 128 samples when calculated SegSNR.

LSD was a measure of the distance of power spectrum between two audios. In this study, it meant the distance of power spectrum between pure feeding sound and denoised feeding sound. The smaller the LSD, the better the denoising effect. The equation of LSD was shown as follows:

(11) $$D_{LS}=\sqrt{\sum {\left[10\times \log_{10}\left(\frac{P_{\text{pure}}}{P_{\text{denoisd}}}\right)\right]}^{2}}$$

where $P_{pure}$ was the power spectrum of pure feeding sound and $P_{denoised}$ was the power spectrum of denoised feeding sound.

### 4.3. Respective Results of TDD-Nets and FDD-Nets

The average ∆SNRs, ∆SegSNRs, and LSDs of TDD-Nets using different convolution blocks are shown in Table 2.

When the convolutions were replaced by dilated convolutions in the convolution blocks, the ∆SNR and ∆SegSNR had significant improvements, which improved by 17.85% and 15.63%, respectively, and the LSD had a significant decline, which fell by 9.38%. That proved that the value of dilated convolutions in extracting the time domain features from the waveform of audio, and when the shortcut was added on the basis of dilated convolutions, the ∆SNR and ∆SegSNR improved by 1.59% and 1.21%, respectively, and the LSD fell by 6.94% compared with the model which only used dilated convolutions. That proved that the shortcut had a positive impact on the denoising effect, although the improvement was not very large, and when the convolution was added in the shortcut, the ∆SNR and ∆SegSNR improved by 3.30% and 2.54% respectively and the LSD fell by 2.30% compared with the model which only used dilated convolutions. That proved that the local correlation between adjacent data extracted by the convolution in the shortcut was helpful for the regression of waveform energy while having some negative effects on the regression of the power spectrum.

The average ∆SNRs, ∆SegSNRs, and LSDs of FDD-Nets using different recurrent layers are shown in Table 3.

When comparing ∆SNR, ∆SegSNR, and LSD in three kinds of recurrent layers, the model that used GRU and LSTM had better denoising effects than that used RNN. Compared to the standard GRU and LSTM with RNN, the ∆SNR improved by 21.77% and 25.59%, the ∆SegSNR improved by 21.65% and 22.47%, and the LSD improved by 36.88% and 40.63%, respectively. Compared to the BiGRU and BiLSTM with BRNN, the ∆SNR improved by 6.16% and 8.61%, the ∆SegSNR improved by 4.60% and 4.42%, and the LSD fell by 23.39% and 32.26%, respectively. The significant performance improvements of GRU and LSTM indicated that the long-term information made an outstanding contribution to frequency domain denoising. Further, the models that used LSTM were better than those that used GRU as the LSTM had better flexibility in selecting information for the training process by using three gates.

When the standard recurrent layers were replaced with the three bidirectional recurrent layers, the ∆SNR and ∆SegSNR had significant improvement while the LSD had a significant decline. The ∆SNR improved by 34.83%, 17.55%, and 16.60%, respectively, the ∆SegSNR improved by 35.65%, 16.63%, and 15.66%, respectively, and the LSD fell by 22.50%, 5.94%, and 11.58% respectively. That results supported the view that the time dependence between the frames in the spectrogram and the time dependence existed not only in the previous time but also in the next time.

In summary, the TDD-Net-DC using the dilated convolution with a convolutional shortcut achieved the best denoising effects in four structures of the TDD-Nets, and the FDD-Net-BL using the BiLSTM achieved the best denoising effects in six structures of the FDD-Nets.

### 4.4. Comparisons between the Best TDD-Nets and FDD-Nets

In order to compare the denoising effects between the TDD-Nets and FDD-Nets, the denoising results of TDD-Net-DC and FDD-Net-BL achieved the best results in the time domain and frequency domain, respectively, and were used for further analysis. At the same time, the wavelet denoising method was selected as the comparison method in the field of traditional denoising methods. The basic function of the wavelet method adopted the db1 wavelet function, the threshold selection algorithm adopted the minimax threshold, and the threshold function adopted the soft threshold function [60].

**Figure 6:**
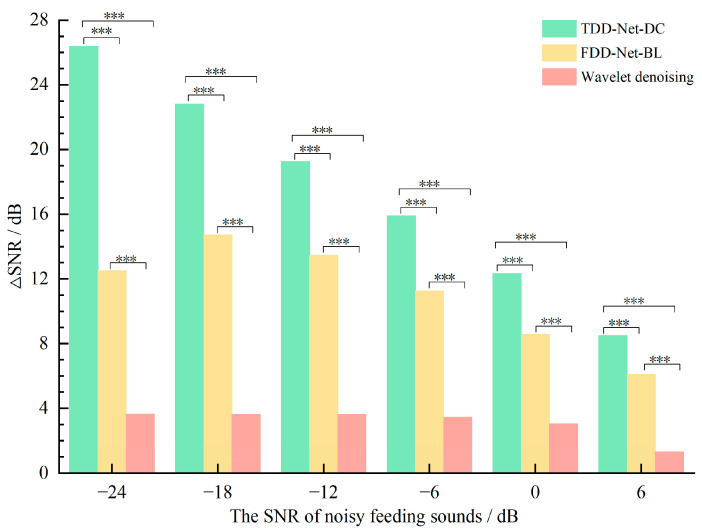
Comparison of the ∆SNRs of different denoising methods at different SNRs, *** means highly significant (*p* ≤ 0.001).

The average ∆SNRs of the three denoising methods are shown in Figure 6. There were denoising effects for all of the three denoising methods at −24 dB to 6 dB noisy feeding sounds.

Among 6 SNRs, the average ∆SNRs of TDD-Net-DC, FDD-Net-BL, and wavelet denoising were 17.53 dB, 11.10 dB, and 3.12 dB, respectively. Compared with the average ∆SNRs of wavelet denoising, that of TDD-Net-DC and FDD-Net-BL improved by 461.86% and 255.77%, respectively. The results of variance analysis showed that ∆SNRs of three denoising methods were significantly different for the test data at each SNR. It could be seen from Figure 6 that the TDD-Net-DC had an obvious denoising effect for the noisy feeding sounds at −24 dB, and the average SNR was increased to 2.38 dB. The average ∆SNR of the FDD-Net-BL at low SNRs was not as good as that of the TDD-Net-DC. However, with the improvement of the SNR of the noisy feeding sounds, the gap in the average ∆SNRs of the two models gradually narrowed, and the average ∆SNR of the FDD-Net-BL reached the peak for the noisy feeding sounds at −18 dB. Although the wavelet denoising method also had a certain denoising effect, the overall denoising effect was weak, which was not more than 4 dB for the improvement at each SNR.

**Figure 7:**
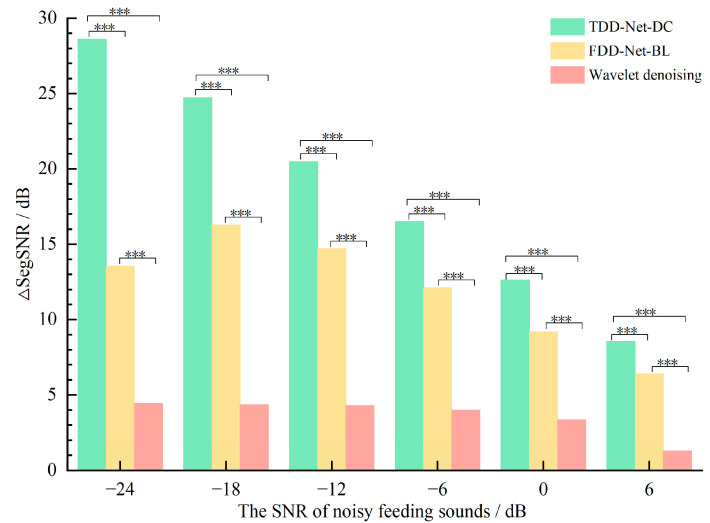
Comparison of the ∆SegSNRs of different denoising methods at different SNRs, *** means highly significant (*p* ≤ 0.001).

The average ∆SegSNRs of the three denoising methods are shown in Figure 7. In the noisy feeding sounds with different SNRs, the changing trend of average ∆SegSNRs was basically the same as that of average ∆SNRs. Among 6 SNRs, the average ∆SegSNRs of TDD-Net-DC, FDD-Net-BL, and wavelet denoising were 18.59 dB, 12.04 dB, and 3.62 dB, respectively. Compared with the average ∆SNRs of wavelet denoising, that of TDD-Net-DC and FDD-Net-BL improved by 413.54% and 232.60%, respectively. The results of variance analysis showed that the ∆SegSNRs of three denoising methods were significantly different for the test data at each SNR. According to the results of average ∆SegSNRs, the TDD-Net-DC had the best denoising effect for the noisy feeding sounds at −24 dB, and the increase reached 28.62 dB, and the FDD-Net-BL had the best denoising effect for the noisy feeding sounds at −18 dB and the increase was 16.28 dB. In contrast, the effect of the wavelet denoising method was relatively general, and the average ∆SegSNR was not more than 5 dB.

**Figure 8:**
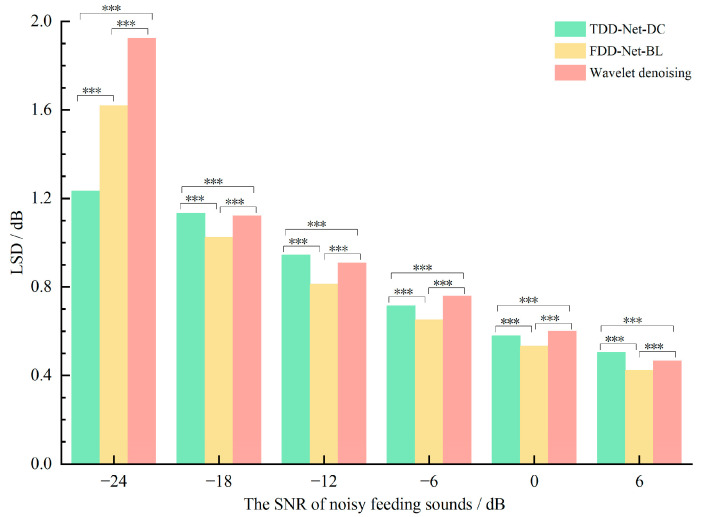
Comparison of the LSD of different denoising methods at different SNRs, *** means highly significant (*p* ≤ 0.001).

The above two metrics, ∆SNRs and ∆SegSNRs, mainly measured the denoising effect from the perspective of signal amplitude. Then, the LSD was used to analyze the denoising effect from the perspective of the spectrum. The comparison of average LSDs between the pure feeding sounds and the denoised feeding sounds denoised by three denoising methods is shown in Figure 8. The smaller the LSD, the higher the spectral similarity between the denoised feeding sounds and pure feeding sounds.

Among 6 SNRs, the average LSDs of TDD-Net-DC, FDD-Net-BL, and wavelet denoising were 0.85 dB, 0.84 dB, and 0.96 dB, respectively. Compared with the average ∆SNRs of wavelet denoising, that of TDD-Net-DC and FDD-Net-BL fell by 11.46% and 12.50%, respectively. The results of variance analysis showed that LSDs of three denoising methods were significantly different for the test data at each SNR. It could be seen from Figure 8 that the denoising effect of TDD-Net-DC was not ideal from the perspective of spectral similarity, expect the noisy feeding sounds at −24 dB. In contrast, the FDD-Net-BL showed great advantages in the spectral similarity, and the average LSD of the wavelet denoising was similar to that of TDD-Net-DC except for the noisy feeding sounds at −24 dB.

### 4.5. Comparisons the Acoustic Detection Effects between Noisy Sound and Denoised Sound

In order to verify the influence of acoustic denoising on the acoustic detection for *S. bifasciatus*, a feeding sounds recognition network (FSRNet) was designed to recognize the *S. bifasciatus* feeding sounds before and after denoising and the denoising results of all three models TDD-Net-DC, FDD-Net-BL and wavelet denoising method were tested.

The FSRNet was trained using three kinds of sounds, which were pure feeding sounds, non-infected sounds recorded in the same place, and environmental noises. There were 13,260 slices with a length of 2 s, 4420 slices for each category. The pure feeding sounds and environmental noises were randomly selected from the train set of the denoising experiment. The architecture of FSRNet is shown in Table 4. Firstly, the spectrum of the sound slice was calculated as the input features of FSRNet. The STFT was performed with a Hann windowing size of 480 samples and a hop size of 160 samples. Then, two convolutional layers with ReLU were used to extract spectral features, and the global average pooling was used to consolidate global features. Finally, a fully connected layer with softmax was used to calculate the classification probability.

**Figure 9:**
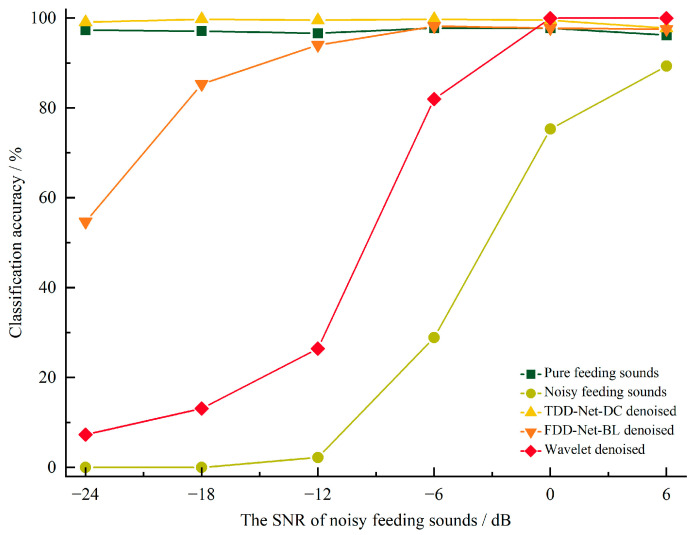
Comparison of classification accuracy for the feeding sounds before and after denoising.

The classification results for different kinds of data are shown in Figure 9. It could be seen that the classification accuracy of noisy feeding sounds decreased significantly with the decrease in SNR, and the classification accuracy of denoised feeding sounds obtained by different denoised methods had been improved to different degrees. The average accuracy of pure feeding sounds, noisy feeding sounds, TDD-Net-DC denoised sounds, FDD-Net-BL denoised sounds, and wavelet denoised sounds were 97.15%, 32.63%, 99.26%, 87.93%, and 54.81%, respectively. The denoised sounds using TDD-Net-DC achieved better classification results compared with the other two methods. It indicated that it removed more distracting information and enhanced enhances the features of feeding sounds when using TDD-Net-DC to denoise.

## 5. Discussion

In this section, the visualization denoising results were shown for the qualitative analysis of the denoising effects of different denoising methods, and the advantages and limitations of the denoising methods were discussed.

**Figure 10:**
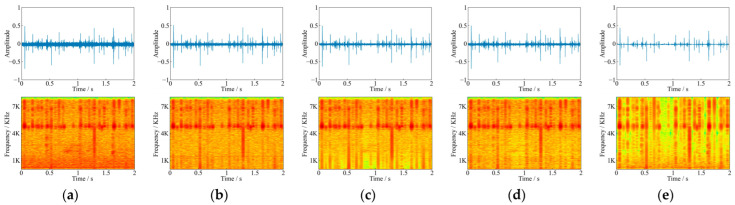
Comparison of waveforms and spectrograms before and after denoising for the noisy feeding sound at 6 dB (**a**) the noisy feeding sound at 6 dB; (**b**) corresponding pure feeding sound; (**c**) the denoising result of TDD-Net-DC; (**d**) the denoising result of FDD-Net-BL; and (**e**) the denoising result of wavelet denoising.

Figure 10 shows the comparison of waveforms and spectrograms before and after denoising for the noisy feeding sound at 6 dB. It could be seen that three denoising methods retained the basic form of pure feeding sound waveform in the time domain, while the wavelet denoising method weakened the energy in low amplitude segments, which resulted in the energy loss of denoised feeding sounds. In the frequency domain, three denoising methods effectively retained the spectral information near 5.0 kHz, and the FDD-Net-BL had more complete spectral information, as shown in Figure 10. The TDD-Net-DC had some spectral information weakening between 0.8–1.0 s, while the wavelet denoising method had obvious spectral information weakening between 0.7–1.1 s and after 1.5 s.

**Figure 11:**
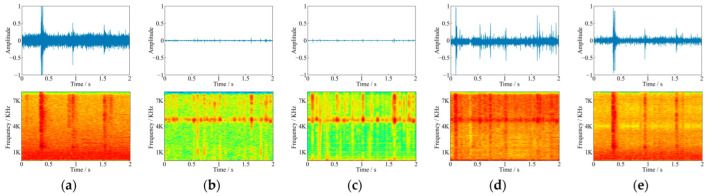
Comparison of waveforms and spectrograms before and after denoising for the noisy feeding sound at −24 dB (**a**) the noisy feeding sound at −24 dB; (**b**) corresponding pure feeding sound; (**c**) the denoising result of TDD-Net-DC; (**d**) the denoising result of FDD-Net-BL; and (**e**) the denoising result of wavelet denoising.

Figure 11 showed the comparison of waveforms and spectrograms before and after denoising for the noisy feeding sound at −24 dB. Three denoising methods weakened the noises in the feeding sound to a certain extent, and the TDD-Net-DC had the best fitting degree to the amplitude intensity. The FDD-Net-BL had an obvious denoising effect on the noise peaks with large amplitude intensity, while the wavelet denoising method only weakened the audio energy as a whole. In the frequency domain, the TDD-Net-DC and FDD-Net-BL had an obvious denoising effect on three obvious noise peaks, and the frequency domain band near 5.0 kHz of pure feeding sound could obviously be seen as shown in Figure 11. However, the results of the wavelet denoising method still retained three noise peaks and did not reflect the spectral information of feeding sound at 5.0 kHz.

Through quantitative and qualitative denoising results analysis, the traditional wavelet denoising method could denoise the noisy feeding sounds of *S. bifasciatus* larvae to a certain extent, but the denoising effect was relatively poor, and the denoised feeding sounds could not reflect the frequency domain features of the feeding sound of pests when the SNR of noisy feeding sounds was low or the noise signals had complex spectral features. When considering the implementation of an algorithm, the wavelet denoising method mainly denoised by filtering the threshold of the decomposition frequency band, which had no training process and could not obtain signal features from pure feeding sounds, and the existing literature using wavelet noise and the similar filtering denoising method almost assumed that the noise was Gaussian noise as the main source of noise in most electronic systems was thermal noise which was typically Gaussian white noise. However, with the change in denoising scenarios and needs, denoising models need to face more complex and variable noise types. The denoising effects of the wavelet and similar principle of denoising methods gradually deteriorated and could not meet the actual demand, such as the acoustic denoising in noisy forests.

Compared with the traditional wavelet denoising method, artificial intelligence methods were more flexible and adaptable in the denoising process. From the principle of the methods, artificial intelligence models had the ability to learn features from data, and they could gradually focus attention on the needed information and ignore the unconcerned content through a supervised iterative process. In the existing literature, the artificial intelligence denoising method was used to extract vocals from music audio signals that considered background accompaniment as noise even if it was actually beautiful music [40]. In the field of speech enhancement, where artificial intelligence methods were widely used, television, music, background chatter, and a series of sounds other than the concerned speaker were all noise [61]. From the existing literature, the artificial intelligence-based noise reduction method was more suitable for the acoustic denoising in noisy forests, and the experimental results showed that the denoising models based on artificial intelligence had achieved better results in the denoising of *S. bifasciatus* larvae feeding sounds and different characteristics were shown in the denoising effects of TDD-Nets and FDD-Nets with the increase in SNRs of noisy feeding sound.

The denoising principle of FDD-Nets was to extract spectral features of feeding sounds for denoising, which could better fit the spectral features. That theoretical analysis was proved according to the comparison of the LSD as shown in Figure 8 and the example of denoising results as shown in Figure 10. In the time domain, as shown in Figure 10 and Figure 11, it could be seen that FDD-Nets could better regress the amplitude energy of feeding sounds at high SNR. However, at low SNR, the amplitude energy of the denoised feeding sounds was obviously high, even slightly higher than that before denoising.

The denoising principle of TDD-Nets was the fitting of feedings sounds waveform. It completely extracted the features of the whole waveform, so the uneven amplitude distribution between samples would have a certain impact on the denoising performance of the model. Therefore, although it could better retain the main waveform and spectral features of feeding sounds, it was not sensitive to samples with low amplitude, which was easy to cause energy loss of denoised feeding sounds in some samples with low amplitude. In addition, since TDD-Nets did not pay attention to the frequency domain features of the feeding sounds, their accuracy in frequency domain regression was relatively general. That theoretical analysis was proved according to the example of denoising results as shown in Figure 10, where TDD-Nets had energy loss at low amplitude segments and had spectral information weakening at some segments.

## 6. Conclusions

In this study, the feeding sounds of *S. bifasciatus* larvae and various environmental noises in a noisy environment were recorded, and the noisy feeding sounds with different SNRs were generated for experiments. Several TDD-Nets using the time domain features and FDD-Nets using the frequency domain features were designed to reduce the noise of noisy feeding sounds with different SNRs and compared with each other. Then the best TDD-Nets and FDD-Nets, TDD-Net-DC and FDD-Net-BL, were selected and further compared with the traditional wavelet denoising method to analyze the denoising mechanism and denoising effect using different methods. The results showed that the average ∆SNRs of TDD-Net-DC, FDD-Net-BL, and wavelet denoising were 17.53 dB, 11.10 dB, and 3.12 dB, respectively, and the average LSD between pure feeding sounds and denoised feeding sounds of TDD-Net-DC, FDD-Net-BL, and wavelet denoising were 0.85 dB, 0.84 dB, and 0.96 dB, respectively.

According to the perspective of signal strength of noises and spectral similarity, the denoising effects of the denoising models based on artificial intelligence were obviously better than that of the wavelet denoising method. Although the denoising effects of FDD-Nets were slightly better on spectral similarity, the overall results of TDD-Nets were better than that of the FDD-Nets. Therefore, the denoising models based on artificial intelligence could effectively denoise the noisy feeding sounds of *S. bifasciatus* larvae, and the TDD-Nets using the time domain features had obvious advantages. For the scene with complex noise environments and large noise interference, the TDD-Nets using the time domain features would have the ability to effectively reduce the noise interference and obtain the relatively pure feeding sounds, and the results of the acoustic detection experiment indicated the proposed methods could remove noise interference and enhance feeding sounds features to a certain extent, which was valuable for the acoustic detection of wood-boring pests in forests. Thus, the proposed methods could obtain purer feeding sounds and lay a foundation for early acoustic detection of wood-boring pests in noisy forests. In the future, the denoising method using time-domain features and frequency domain features at the same time would be designed to integrate the advantages of time-domain denoising and frequency domain denoising to achieve a better denoising effect. In addition, the real noisy feeding sounds in forests would be recorded to further test and optimize the denoising models.

## Figures and Tables

**Table 1:** The statistics of the dataset.

Category	Training		Test	
	Audio Segments	Audio Slices	Audio Segments	Audio Slices
*S.bifasciatus* feeding sounds	42	6300	18	2700
Environmental noises	42	6300	18	2700
Total	84	12,600	36	5400

**Table 2:** Comparison with TDD-Nets using different convolution blocks. The red numbers were the best results of the corresponding items.

Models	Average ΔSNR (dB)	Average ΔSegSNR (dB)	Average LSD (dB)
TDD-Net-C	14.40	15.68	0.96
TDD-Net-D	16.97	18.13	0.87
TDD-Net-DS	17.24	18.35	0.81
TDD-Net-DC	17.53	18.59	0.85

**Table 3:** Comparison with FDD-Nets using different recurrent layers. The red numbers were the best results of the corresponding items.

Models	Average ΔSNR (dB)	Average ΔSegSNR (dB)	Average LSD (dB)
FDD-Net-R	7.58	8.50	1.60
FDD-Net-G	9.23	10.34	1.01
FDD-Net-L	9.52	10.41	0.95
FDD-Net-BR	10.22	11.53	1.24
FDD-Net-BG	10.85	12.06	0.95
FDD-Net-BL	11.10	12.04	0.84

**Table 4:** The architecture of FSRNet.

#	Layer	Parameters	Activation
1	Convolution (3 × 3)	Filter size = 32, Stride = 2	ReLU
2	Convolution (3 × 3)	Filter size = 64, Stride = 2	ReLU
4	Global Average Pooling		
5	Fully connected	Size = 3	Softmax

## Data Availability

Not applicable.
